# PROTOCOL: The effectiveness of sensory interventions targeted at improving occupational outcomes, quality of life, well‐being and behavioural and psychological symptoms for older adults living with dementia: A systematic review and meta‐analysis

**DOI:** 10.1002/cl2.1322

**Published:** 2023-04-10

**Authors:** Nikki Tulliani, Caroline Mills, Lily Collison, Nicole Peel, Paul P. Fahey, Karen Liu

**Affiliations:** ^1^ School of Health Sciences Western Sydney University Campbelltown Australia; ^2^ Translation Health Research Institute Western Sydney University Campbelltown Australia

## Abstract

This is the protocol for a Campbell systematic review. The objectives are as follows: The primary objective is to systematically review the available evidence of the effects of sensory interventions on quality of life, well‐being, occupational participation, and behavioural and psychological symptoms of older adults living with dementia.

## 1 BACKGROUND

### 1.1 The problem, condition or issue

Dementia is a chronic and progressive syndrome in which there is deterioration in cognitive function greater than that commonly expected as part of the ageing process (World Health Organization, 2004). Dementia is currently one of the major causes of disability and dependency among older people worldwide (World Health Organization, 2004). In 2021, more than 55 million people were living with dementia globally. This number is predicted to reach 78 million in 2030 and a staggering 139 million by 2050 (World Health Organization, 2004).

A person with dementia may experience impairment in cognitive functions such as memory, thinking, orientation, comprehension, calculation, learning capacity, language, and judgement (World Health Organization, 2004). These impairments in cognitive functions are commonly accompanied by changes in mood, emotional control, behaviour, or motivation (World Health Organization, 2004) and are commonly referred to as Behavioural and Psychological Symptoms of Dementia (BPSD). BPSD occur in approximately 90% of patients with dementia (Chiu et al., 2006) and includes symptoms such as wandering, verbal aggression, anxiety, agitation, irritability, aberrant motor behaviour, night‐time/sleep disturbances, psychosis, depression, disinhibition and apathy (Petrovic et al., 2007).

Managing BPSD is one of the most significant challenges in providing care to people with dementia (Feast et al., 2016) and can lead to poor health and well‐being outcomes for the person with dementia and for those providing the care (Fogg et al., 2018; Hessler et al., 2018; Tropea et al., 2017). People with dementia who are hospitalised are vulnerable (Tropea et al., 2017) and may require additional care strategies to be implemented to ensure optimal care can be provided during their hospital stay (Fogg et al., 2018).

### 1.2 The intervention

Sensory interventions encompass a range of different types of activities with varying senses utilised along with differences in duration, intensity and frequency. Interventions may include targeted clinic‐based remedial sensory integration therapy (Schaaf et al., 2018), the use of sensory activities embedded in everyday contexts (Mills et al., 2021), psycho‐acoustic/music therapy (Gee et al., 2014; LaGasse et al., 2019), the use of multi‐sensory environments (Breslin et al., 2020; Cameron et al., 2020) and Snoezelen rooms (Lotan & Gold, 2009). These interventions have been implemented in a wide range of settings, age groups, disability diagnoses and with varying desired outcomes making clear comparisons between studies difficult. There is little consistency in current literature about the best way to implement sensory interventions nor is there consensus on the most appropriate dosage (Pfeiffer et al., 2018). For the purposes of this review, studies will be included if they seek to stimulate any of the internal or external senses such as through music or movement, or studies that modify the sensory environment or sensory aspects of an activity such as through the use of sensory items or sensory rooms. Considering the variation in reported interventions, this review will seek to include detailed information about intensity, mode of delivery, frequency, duration and timing of delivery to study the interventions in detail.

### 1.3 How the intervention might work

Sensory interventions are often indicated for those with sensory processing difficulties to alleviate the negative impact of such difficulties on behaviour and functioning in everyday life (Pfeiffer et al., 2018). Sensory interventions are also proposed to mitigate or remediate underlying difficulties a person has with sensory processing (Camarata et al., 2020). Models of sensory processing have suggested that some individuals respond to sensory inputs sooner and more intensely, while others may respond to sensations later and may miss important environmental cues which may negatively impact function and participation in daily activities (Camarata et al., 2020). From this, interventions may attempt to remediate a person's individual difficulties by supporting them to cope with more intense sensations or to more successfully tune into sensations within the environment (Camarata et al., 2020). Conversely, some scholars have proposed that sensory processing does not sit solely within an individual, but rather difficulties experienced are also related to the activity and the environment (Dunn et al., 2016). Interventions may focus on modifications to the activity or the environment to address sensory processing difficulties (Dunn et al., 2016). A literature gap currently exists with respect to the best way to successfully implement sensory interventions (Pfeiffer et al., 2018) and with most of the literature focused on children (Bodison & Parham, 2018) there is no clear answer in terms of the best approach to support older adults.

### 1.4 Why it is important to do this review

Hospitalisation rates are higher among older people with dementia than among older people without dementia (Shepherd et al., 2019). However, a broad range of evidence suggests that hospitals are not good places for people with dementia (Casafont et al., 2022; Hessler et al., 2018; Ní Chróinín et al., 2021) with one‐third of people with dementia being discharged from hospitals with reduced functional capacity in comparison to pre‐admission capacity (World Health Organization, 2018).

During hospitalisation, the combination of an unfamiliar environment and attempts to effectively process the associated sensory information can have a negative impact on people living with dementia and exacerbate the BPSD. These behaviours may impact the care provided and the hospital experience for people with dementia and their caregivers. Increased risk of falling, functional decline, disorientation, poor nutrition and hydration, increased dependence on care givers, depression and delirium are some of the consequences of hospitalisation (Archibald, 2006; Ayton et al., 2017; Bezzant, 2008; Draper et al., 2011; Jurgens et al., 2012).

Earlier studies by Baker et al. (2001) and Maseda et al. (2014) have demonstrated sensory interventions to be beneficial for older adults living in residential aged care. However, there is currently no research exploring the benefits of sensory interventions in a hospital acute care setting. To develop appropriate sensory interventions to support and improve the hospital experience for older people with dementia, it is important to first understand what sensory interventions are being used, both within and outside of acute care settings and if they are beneficial for occupational outcomes, quality of life, well‐being, and BPSD.

If sensory interventions are found to be beneficial, the results of this systematic review will be used to inform the development of an acute care sensory‐based intervention. Furthermore, the results may support the up skilling of clinicians through an evidence‐based approach to support older adults with BPSD not only in acute care settings, but also in the community and residential aged care facilities. Further to this, if the results of this review support sensory interventions for reducing BPSD and improving well‐being and occupational performance for older adults with dementia, this may impact policies and procedures relating to (1) staff allocation (occupational therapist, recreational therapist, music therapist, etc); (2) time allocation and priority of service; (3) staff training; (4) allocation of equipment to support the use of sensory interventions within hospital settings; and (5) a model of care for all people with dementia who are hospitalised.

## 2 OBJECTIVES

The primary objective is to systematically review the available evidence of the effects of sensory interventions on quality of life, well‐being, occupational participation, and behavioural and psychological symptoms of older adults living with dementia.

Where possible we will synthesise the evidence to answer the following research questions:

1. What types (settings, mode, frequency and duration) of sensory interventions are being used with older adults with dementia?
2. What is the effectiveness of sensory interventions versus standard care in maintaining or improving occupational outcomes for people with dementia?
3. What is the effectiveness of sensory interventions versus standard care in maintaining or improving quality of life and well‐being for people with dementia?
4. What is the effectiveness of sensory interventions versus standard care in reducing the behavioural and psychological symptoms of dementia?

## 3 METHODS

### 3.1 Criteria for considering studies for this review

#### 3.1.1 Types of studies

This review will consider level 1 randomised control trials (RCTs) to be eligible. Case controls, crossover trials, one‐arm trials, non‐randomised trials, cross‐sectional studies, and cohort studies will be excluded from this review. Previous level 1 systematic reviews will be excluded from this review but each of their component papers will be reviewed for inclusion. Should there be insufficient randomised trials identified (based on the optimal information size of a total of 400 participants in each control and intervention group will be required [Guyatt et al., 2011]), consideration will be given to including level 2 studies. Experimental non‐randomised control trials (quasi‐experimental with control group and pre‐ and post‐data) will be included as level 2 studies. If there are still too few studies available, level 3 observational cohort studies (with control group and pre‐ and post‐data) will be included. The level of evidence is in accordance with the Johns Hopkins hierarchy of evidence (Dang et al., 2021). This review will adopt an index date of 1979. Dr Jean Ayres was a luminary in the field of sensory interventions. Her seminal work was published in 1979. For this reason, this has been chosen as the index date (Ayres, 2005).

#### 3.1.2 Types of participants

Studies with older people as participants (60+ years), residing in either the community, within a residential aged care setting or within an acute hospital setting and with a diagnosis of dementia as outlined by one of the following criteria, will be included:

- World Health Organisation's International Classification of Diseases code (World Health Organization, 2022)
- National Institute of Neurological and Communicative Disorders and Stroke and the Alzheimer's Disease and Related Disorders Association criteria (Mckhann et al., 1984)
- American Psychiatric Association's Diagnostic and Statistical Manual of Mental Disorders (American Psychiatric Association, 2013)
- Clinical Dementia Rating Scale (Hughes et al., 1982)
- Blessed Dementia Rating Scale (Blessed et al., 1968)
- National Institute on Aging and the Alzheimer's Association (Albert et al., 2011)

Studies reporting participants with dementia utilising an alternative diagnostic criterion will be considered if the diagnostic criteria are standardised, valid and reliable.

Studies that also include younger adults (<60 years) will only be included if (a) they report the results separately for participants 60 years and above; or (b) they specifically define the population as ‘older people’ or ‘older adults’ or ‘elderly’ and the mean age of participants is reported to be greater than 60 years.

Studies of adults with a diagnosis of mild cognitive impairment (MCI), or where dementia is not the primary diagnosis, will be excluded. Participants with common co‐occurring conditions like depression will not be excluded. Corresponding authors will be contacted if clarification to determine health status is required. If identified studies include mixed cohorts (including healthy adults, MCI or dementia, or combining with people younger than 60 years), an attempt will be made to contact the corresponding author to request results for just the eligible participants. If not, these studies will be excluded from the review.

#### 3.1.3 Types of interventions

**Figure 1:**
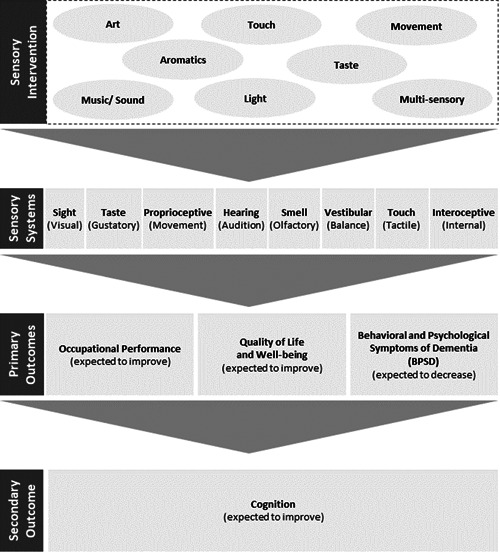
Logic model of how sensory intervention may lead to positive outcomes for people with dementia.

This review will consider studies that use sensory interventions that seeks to stimulate any of the internal or external senses to achieve a therapeutic outcome. A logic model showing the link between the interventions and the outcomes is shown as Figure 1. Sensory interventions may be targeted at one of the following senses: visual, auditory, olfactory, gustatory, tactile, vestibular, proprioceptive or interoceptive. Examples of interventions include music or movement interventions or modifications to the sensory environment such as using sensory rooms. Modes of intervention delivery may comprise face‐to‐face, computer‐administered, individual, or group interventions. Interventions delivered in any setting, inclusive of inpatient and outpatient hospital settings, community‐based programmes, rehabilitation settings, adult day support facilities, and residential aged care facilities, will be included.

Studies looking at the effects of sensory intervention combined with another intervention type will be excluded, such as for example a sensory intervention combined with cognitive behaviour therapy, unless the added intervention was provided in a standardised manner to both experimental and control groups. Studies that do not specify the type of sensory intervention administered will also be excluded.

#### 3.1.4 Types of outcome measures

Studies will be included if they reported the effect of a sensory intervention on at least one of the primary or secondary outcomes. If outcome data is missing from a study, the authors will be contacted before exclusion to determine whether the data for our outcomes of interest are unavailable to lack of reporting and if the data can be made available for this review.

##### Primary outcomes

The primary outcomes are occupational performance, well‐being, quality of life and BPSD (See Figure 1). Outcomes can be assessed by a health care professional, self‐reported by the participant, or informant reported by a caregiver or significant other. Both standardised and non‐standardised assessments will be included.

For the purpose of this review, occupational performance is the accomplishment of one or more selected occupations resulting from the dynamic transaction among the person, their environment, and the occupations (Boop et al., 2020). Occupations will be considered if they fall within one of the following nine categories as activities of daily living (ADLs), instrumental activities of daily living (IADLs), health management, rest and sleep, education, work, play, leisure, and social participation (Boop et al., 2020). Studies that have measured occupational outcomes such as occupational performance, occupational engagement or occupational participation will be included, regardless of the measurement tool used. Measurement tools include, but are not limited to:

- Canadian Occupational Performance Measure (COPM) (Law et al., 1990)
- Functional Independence Measure (FIM™) (Wright, 2011)
- The Barthel Index/Modified Barthel Index (Mahoney & Barthel, 1965)
- The Routine Task Inventory (RTI) (Albert et al., 2011; Allen et al., 1992)
- The Kohlman Evaluation of Living Skills (KELS) (Thomson, 1992)
- Lawton's Instrumental Activities of Daily Living Scale (Lawton & Brody, 1969)
- The Occupational Circumstances Assessment Interview and Rating Scale (OCAIRS) (Deshpande et al., 2002)
- The University of California Performance‐Based Skills Assessment (Gomar et al., 2011)
- The Model of Human Occupation Screening Tool (MOHOST) (Parkinson et al., 2006)

In this review, Studies that have measured wellbeing or quality of life (e.g., life satisfaction, subjective happiness, quality of life, self‐esteem, positive feelings, pain and discomfort, energy and fatigue) will also be included. Due to a lack of a consensual definition for quality of life and the multi‐dimensional nature of the concept (The WHOQOL Group, 2012), the authors expect dimensions of ‘quality of life' and ‘well‐being' to overlap. This review will define quality of life as a broad concept which aims to capture the well‐being of an individual or group of people. In line with the World Health Organisation's definition, quality of life incorporates a persons' subjective experience of their physical health, psychological state, level of independence, social relationships, personal beliefs and their relationships to their context (The WHOQOL Group, 2012).

Measurement tools include, but are not limited to:

- The Psychological Wellbeing Scale (Ryff, 1989) or its shortened version (Ryff & Keyes, 1995; Ryff, 1989)
- 54‐item Comprehensive Inventory of Thriving (CIT) (Su et al., 2014)
- The 22‐item General Well‐being Schedule (Dupuy, 1977)
- The Life Satisfaction Index A (LSIA) (Neugarten et al., 1961)
- The satisfaction with life scale (Diener et al., 1985)
- The Hope Scale (Snyder et al., 1996)
- The CASP‐19 Quality of Life Scale (Hyde et al., 2003)
- The EuroQol 5 Dimensions scales (EQ‐5D)(The EuroQol Group, 1990)

In this review, BSPD represent a heterogeneous group of non‐cognitive and non‐neurological symptoms and behaviours associated with a diagnosis of dementia, such as agitation, aggression, wandering behaviour, sleep disturbances psychosis, depression, and apathy (Cerejeira et al., 2012). Studies that have measured the behavioural and psychological symptoms of dementia (e.g., restlessness, agitation, aggression, psychosis, wandering behaviour, sleep disturbances, depression/dysphoria) will be included. Measurement tools include, but are not limited to:

- Neuropsychiatric Inventory (NPI)(Cummings et al., 1994)
- The Behavioral Pathology in Alzheimer's Disease rating scale (BEHAVE‐AD) (Reisberg et al., 1997)
- The Cohen‐Mansfield Agitation Inventory (CMAI)(Cohen‐Mansfield et al., 1989)
- Consortium to Establish a Registry for Alzheimer's Disease‐Behavior Rating Scale for Dementia (CERAD‐BRSD) (CERAD, 1997)
- The Dementia Behavior Disturbance Scale (Baumgarten et al., 1990)
- Neurobehavioral Rating Scale (Levin et al., 1987)
- The Pittsburgh Agitation Scale (PAS)(Rosen et al., 1994)

##### Secondary outcomes

The secondary outcome of interest is cognition. For the purpose of this review, cognition is defined as the complex mental and intellectual processes such as attention, memory, sensory processing, emotional regulation, motor control and planning, required for thinking, reasoning and problem solving (Gillen, 2019). Data will be extracted from studies that administered a valid and reliable cognitive test pre‐ and post‐sensory intervention, that measured any cognitive domain, including but not limited to episodic memory, executive functioning, attention/working memory, and verbal fluency.

#### 3.1.5 Duration of follow‐up

This systematic review will include studies where the outcome measure of interest has been conducted at least once at baseline (prior to sensory intervention) and once post‐intervention period (either immediately after the sensory intervention or as a long‐term follow‐up).

#### 3.1.6 Types of settings

We will consider sensory interventions executed in any country and will apply no limits on the setting (e.g., hospital, residential aged care, community).

### 3.2 Search methods for identification of studies

A comprehensive search for eligible published and unpublished studies and reports will be performed to reduce the risk of publication bias and identify the best available evidence. Only studies in the English language will be included in the review. No other limitations will be used during the database searches.

#### 3.2.1 Electronic searches

The following databases will be searched from inception to present:

- OVID Medline
- OVID EMBASE
- EBSCO version of CINAHL
- EBSCO version of PsycInfo
- Scopus
- Central Register of Controlled Trials
- Social Science Citation Index (via Web of Science)
- ASSIA
- AgeLine (by EBSCO)

The search strategy was developed by NT and CM in consultation with a health sciences librarian (LC). The search strategy aims to locate both published and unpublished studies. A three‐step search strategy is underway for this review. First an initial limited search of OVID SP versions of MEDLINE and EMBASE using free text terms has been undertaken to identify articles on the topic. Text words contained in the titles and abstracts of relevant articles as well as index terms used to describe the articles supplemented with additional index terms located in the database thesaurus were used to develop a full search strategy.

The search will be tailored to thesaurus or controlled‐vocabulary and search syntax of each database and will comprise both index terms (when relevant; e.g., MeSH terms) and free text words (in title or abstract) with attention to possible synonyms, spelling variants, and correct use of truncation and proximity operator. Search filters will not be used, as they may prevent the retrieval of relevant papers. The full search strategy for MEDLINE (OVID SP version) is provided in the Supporting Information: Appendix 1.

To ensure that the search strategy is comprehensive and overcomes publication bias, Trial registry database will be monitored for high‐quality studies that are underway and nearing completion/publication and to capture findings with null results which may not be published.

Trial registry databases:

- ClinicalTrials.gov (https://clinicaltrials.gov/)
- International Clinical Trials Registry Platform of the World Health Organization (https://trialsearch.who.int/)
- Australian New Zealand Clinical Trials Registry (https://anzctr.org.au/)
- ISRCTN registry (https://www.isrctn.com/)

Internet searches:

Google Scholar (https://scholar.google.com/) will also be used to search for published level 1 studies. A subset of terms from the search strategy will be used with the following search string to be used in Google Scholar ‘dementia, sensory, BPSD, occupation, Quality of Life, Well‐being site:org’. Publish or Perish software (Harzing, 2007) will be used to download the first 1000 results from Google Scholar. A limit will be placed on the number of items to screen as search engines yield an unmanageable number of results with diminishing relevance down the results pages (Rethlefsen et al., 2021; Stansfield et al., 2016). Only a title search will be conducted because searching by title is more efficient than searching the full text in Google Scholar for reviews (Haddaway et al., 2015; Haddaway et al., 2017). To reduce the risk of personalisation bias, Google and Google scholar searches will be conducted in incognito mode.

Websites:

- WHO Ageing and life‐course Program (www.who.int/ageing/data-research/en/)
- National Ageing Research Institute (NARI), Australia (www.nari.net.au/publications/overview-about-publications)

Centres and research groups attached to an academic institution will not be included in the grey literature search, as it is expected that they publish their work in peer‐reviewed journals and relevant studies will be discovered in the electronic database searches.

#### 3.2.2 Searching other resources

##### Other reviews

Citation checking will be carried out on any relevant systematic reviews identified using the search above search to identify any additional studies missed by the database search.

##### Reference lists

Citation tracking will be carried out on all articles identified in the final data analysis group to identify any additional studies missed by the database search.

##### Handsearching of journals

Handsearching using dementia, sensory, BPSD, occupation, Quality of Life, Well‐being will be conducted on the top 5 most cited journals identified in the final data analysis group of records.

##### Contact to experts

We will contact international experts to identify unpublished or ongoing studies.

### 3.3 Data collection and analysis

#### 3.3.1 Description of methods used in primary research

Briefly describe the anticipated methods that included studies are likely to employ.

#### 3.3.2 Selection of studies

Following the search, all identified citations will be collated and uploaded into EndNote X8 reference management system (Clarivate Analytics, 2016) and then uploaded to Covidence systematic review platform (Covidence systematic review software, 2019) and duplicates removed. Following a pilot test of 10% of the identified studies, titles and abstracts will then be screened independently and in parallel by two or more independent reviewers (NT, CM, NP) for assessment against the inclusion criteria for the review. Potentially relevant studies will be retrieved in full. The full text of selected studies will be assessed in detail against the inclusion criteria by two or more independent reviewers (NT, CM, NP) and references that meet the selection criteria will be included for further analysis. Reasons for exclusion of papers at full text that do not meet the inclusion criteria will be recorded and reported in the systematic review. Any disagreements that arise between the reviewers at each stage of the selection process will be resolved through discussion until consensus is reached, and in case of disagreement an additional reviewer (KL) will be involved. The results of the search and the study inclusion process will be reported in full in the final systematic review and presented in a Preferred Reporting Items for Systematic Reviews and Meta‐analyses (PRISMA) flow diagram for new systematic reviews which included searches of databases, registers and other sources (Page et al., 2021).

The searches will be re‐run just before final analyses and further studies retrieved for inclusion if found. All searches and search dates will be documented and reported.

#### 3.3.3 Data extraction and management

Data will be extracted from studies included in the review by two reviewers working independently (NT, CM or NP). To ensure consistency in the data collection process a modified version of the Cochrane data collection form (CDCF) for intervention reviews for RCTs (Cochrane Collaboration, 2014) will be used. If insufficient RCTs are identified during the search, a modified version of the CDCF for intervention reviews for RCTs and non‐RCTs (Cochrane Collaboration, 2014) will be used.

The following data will be extracted where available:

1. Participant information: gender, mean age, educational level, ethnicity, diagnosis and diagnostic criteria utilised, and baseline cognitive score if indicated.
2. Methods of each study: Study design, sample size, treatment setting, relevant outcome measure/s, duration of follow‐up, country of origin, source of financial support and methodological limitations reported.
3. Type of interventions: Aim of intervention, type and description of sensory intervention and control group, duration of treatment (duration of sessions, frequency of sessions, period of intervention, total hours of intervention) and method of intervention delivery (individualised or group).
4. Outcome measures: pre‐intervention (if indicated) and post‐intervention outcome scores or overall change in outcome score, and follow‐up if data is available.
5. Results of the studies: Effect size for experimental and control groups.

Any disagreements between the reviewers will be resolved through discussion or with a third reviewer (KL). Authors of papers will be contacted via email to request missing or additional data, where required.

#### 3.3.4 Assessment of risk of bias in included studies

Each study included in the review will be assessed for risk of bias, by the two independent reviewers (CM, NT or NP). Any disagreements that arise between the reviewers will be resolved through discussion or with a third reviewer (KL). The Cochrane Risk of Bias tool will be used to identify the methodological quality for RCTs (Higgins, Savović, et al., 2019). If non‐RCTs are included, risk of bias will then be assessed using the Risk of Bias in Non‐randomized Studies‐of Interventions (ROBINS‐I) (Sterne et al., 2016; Sterne et al., 2019). Authors of papers will be contacted for clarification, where required.

#### 3.3.5 Measures of treatment effect

Each study and outcome measure will be assessed for suitability for meta‐analysis. Two reviewers (NT, CM or NP) will evaluate and identify the major outcome measure that represents the main outcome of each study for meta‐analysis. If multiple methods are used to measure the same outcome within a study, the reviewers will extract information from all outcome measures to report in the narrative analysis. Where included papers present more than one valid and reliable measurement of a single outcome of interest, robust variance estimation techniques implemented through the robumeta() command in R software will be used in the meta‐analysis. The *I* ^2^ and tau measures of heterogeneity will be interpreted as close approximations (Tanner‐Smith et al., 2016).

The treatment effects, based on pooled data from individual studies, will be recorded. Means and standard deviations (SDs) or medians at pre‐, post‐intervention and follow‐up assessments will be extracted from each study. If the means and SDs or medians are not available, the corresponding author will be contacted for the available data. If further information is not available, medians will be used to replace means, and baseline SD will be used as an estimate of SD at follow‐up. If the required data cannot be retrieved, the study will be excluded from the meta‐analysis.

#### 3.3.6 Unit of analysis issues

In RCT studies, each study will be assessed to determine how and when randomisations occurred. This will be taken into account when considering whether participants were randomised in a cluster or group or whether they were randomised individually. The sample size will be weighted downwards by the estimated design effect. When a a clustered RCT design has been used, without appropriate analyses for the design, the design effect will be estimated using the intra‐class correlation coefficient (ICC) if provided. If the ICC is not provided it will be estimated from the wider literature. The effect size of individual outcome measures will be calculated using Hedge's *g*, with included adjustments for small sample size. The analysis will be performed using Review Manager (RevMan) (Review Manager Web, 2020) where the random effect model with 95% confidence interval (CI) will be used.

If a study compared the effects of sensory interventions across two treatment groups on the outcome relative to the control, the two treatment groups will be included using robust variance estimation techniques (Hedges et al., 2010). If a study includes a treatment group not targeting the outcome of interest of interest (i.e., falls reduction), it will be used as the control group or not included in the analysis. Any treatment targeting ‘occupational outcomes’, ‘quality of life and wellbeing’, ‘behavioural and psychological symptoms’ and ‘cognition’ will be included in analysis group. If a post‐intervention score is not available for an outcome measure of interest after contacting the corresponding author via email, the study will be excluded from the analysis.

#### 3.3.7 Criteria for determination of independent findings

As previously mentioned, treatment setting will be recorded for each study. As there are large numbers of people with dementia, it is unlikely that studies conducted in different settings will contain the same individuals. Any studies conducted in the same setting will be reviewed for possible overlaps.

#### 3.3.8 Dealing with missing data

If correspondence details are available, authors of studies will be contacted via email to request missing or additional data, where required. If the information is not available after this process, where possible, the most conservative estimates will be made using available data (e.g., risk ratios, 95% CI and *p* values) (Higgins, Li, et al., 2019), using the Review Manager 5 software (Review Manager Web, 2020). If insufficient data are available to calculate missing values, only narrative analysis will be performed for these studies.

#### 3.3.9 Assessment of heterogeneity

Heterogeneity will be assessed using *I* ^2^ and *τ* ^2^ statistics. Meta‐analysis with an *I* ^2^ between 50% and 90% will be considered to have substantial heterogeneity and appropriate warnings will be given against over interpretation of these results (Deeks et al., 2022).

#### 3.3.10 Assessment of reporting biases

A funnel plot will be generated using RevMan (RevMan Web, 2020) to assess publication bias if there are 10 or more studies included in a meta‐analysis (Sterne et al., 2011). Initially publication bias will be assessed through visual examination of funnel plots and statistical tests for funnel plot asymmetry (Egger test) will be performed where appropriate. If there is evidence of funnel plot asymmetry following the Egger test, we will attempt to explain possible reasons for bias such as poor methodological quality leading to false inflated effects in smaller studies, nonreporting biases, true heterogeneity.

#### 3.3.11 Data synthesis

The results of clinically and statistically homogeneous studies will be meta‐analysed using RevMan (RevMan Web, 2020). Meta‐analysis will be conducted using the Mantel–Haenszel method for dichotomous outcomes, and the inverse variance method for continuous outcome. A random‐effects model will be used. Where meta‐analysis is not possible, findings will be presented in narrative form including tables and figures to aid in data presentation. Synthesis of findings will be presented in accordance with one of the alternative synthesis and visual display methods as described in the Cochrane Handbook for Systematic Reviews of Interventions (McKenzie et al., 2019).

Forest plots will be inspected to visually investigate overlap in the confidence intervals for the results of the individual studies. Heterogeneity will be assessed statistically using the standard *χ* ^2^ and *I* ^2^ tests. For the *χ* ^2^ test, a *p* value of 0.10 will be used as a threshold for statistical significance.

#### 3.3.12 Subgroup analysis and investigation of heterogeneity

Subgroup analyses will be conducted where there is sufficient data to investigate.

A subgroup analysis of the effect of different types of sensory interventions on occupational outcomes, well‐being, quality of life, and BPSD will be conducted to determine the sensory intervention with greater effect size. The interventions will be categorised into eight groups: visual, auditory, olfactory, gustatory, tactile, vestibular, proprioceptive and interoceptive. Studies that combine two different sensory interventions will be excluded from this analysis.

A subgroup analysis of the effect of sensory interventions using internal sensory systems such as movement and body position and interventions using external sensory systems such as interventions targeting auditory, vision or touch input on occupational outcomes, well‐being and quality of life, and BPSD will be conducted to determine the sensory intervention with greater effect size. Studies that combine sensory interventions using both internal and external sensors will be excluded from this analysis.

If there is sufficient data available, a subgroup analysis of the severity of dementia (mild, moderate, severe) will be conducted to determine if sensory interventions are more effective and beneficial to people with a mild, moderate or severe dementia diagnosis. Studies will be included if participants have been classified according to the severity of dementia utilising a standardised, valid, and reliable diagnostic criterion. Studies that do not report the severity of dementia or combine participants with varying severities of dementia will be excluded from this analysis.

If there is sufficient data within studies which included participants with common co‐occurring conditions such as depression and/or anxiety, a subgroup analysis will be conducted for each condition. This will aim to determine whether sensory interventions are more or less effective for people with dementia and co‐occurring diagnoses compared with dementia alone.

A subgroup analysis of the setting type (e.g., residential aged care, community, hospital) will be conducted to determine if the setting type influences the effect size of sensory intervention. A subgroup analysis for frequency and duration of intervention will also be conducted. A final subgroup analysis examining group intervention and individualised intervention will also be performed. If any additional subgroup analyses are conducted, it will be clearly outlined in the review that these were conducted post hoc and were exploratory in nature.

All subgroup analysis will be performed using Review Manager 5 software (Review Manager Web, 2020). Effect sizes of 0.2, 0.5 and 0.8 represent small, moderate and large effects, respectively (Cohen, 2013). The statistical heterogeneity of the studies will be evaluated using the *I* ^2^ statistic. Random effects models will be used, as the estimated effects in the included studies are unlikely to be identical. Meta‐analysis with an *I* ^2^ between 50% and 90% will be considered to have substantial heterogeneity (Deeks et al., 2022). The funnel plot asymmetry test to distinguish chance from real asymmetry will be applied if there is sufficient power (greater than 10 studies included in the subgroup analysis (Sterne et al., 2011).

If any additional subgroup analyses are conducted, it will be clearly outlined in the review that these were conducted post hoc and were exploratory in nature.

If enough studies are identified, meta‐regression will be conducted using Metareg meta‐regression package in R statistical software (Viechtbauer, 2010), as this is not possible in the Review Manager 5 software (Review Manager Web, 2020).

#### 3.3.13 Sensitivity analysis

We will not separate the meta‐analyses by study design; however, the robustness and generalisability of the results will be explored by a variety of sensitivity analyses such as excluding the lower quality studies and studies from less developed or developing countries. The sensitivity analyses will assist to determine if the results were influenced by the inclusion or exclusion of lower‐quality studies. An experienced statistician (PF) will assist with the completion of the meta‐analysis and sensitivity analysis.

#### 3.3.14 Treatment of qualitative research

We do not plan to include qualitative research.

#### 3.3.15 Summary of findings and assessment of the certainty of the evidence

In the full review, we will provide summary of findings tables and an assessment of the certainty of the evidence based on the included studies.

The Grading of Recommendations, Assessment, Development and Evaluation (GRADE) (GRADE Working Group, 2012) approach will be used to assess the overall certainty of the evidence included in this review and a ‘summary of findings’ table will be created using GRADEpro GDT (GRADEpro GDT, 2015). This will be undertaken by two independent reviewers at the outcome level (KL, CM, NT or NP). Any disagreements that arise between the reviewers will be resolved through discussion or with a third reviewer. The certainty of the ‘body of evidence’ included in this review will be assigned, ranging from high, moderate, low to very low.

The summary of findings table will present the following information where appropriate: absolute risks for the treatment and control, estimates of relative risk, mean differences, standardised mean differences and a ranking of the quality of the evidence based on the risk of bias, directness, heterogeneity, precision and risk of publication bias of the review results.

## CONTRIBUTIONS OF AUTHORS

- Content: Nikki Tulliani, Caroline Mills, Nicole Peel
- Systematic review methods: Karen Liu, Paul Fahey
- Statistical analysis: Paul Fahey, Karen Liu
- Information retrieval: Lily Collison

## DECLARATIONS OF INTEREST

The authors of this systematic review and meta‐analysis have no conflicts of interest.

## PLANS FOR UPDATING THIS REVIEW

The systematic review and meta‐analysis will be updated by atleast one member of the research team every 3 years.

## PRELIMINARY TIMEFRAME

It is expected that this systematic review will be completed by mid to late 2023.

## SOURCES OF SUPPORT

**Internal sources**

- None, Australia
  There is no internal sources of support.
**External sources**
- None, Australia

There is no external sources of support.

## Supporting information

Supporting information.
