# Supplementary material for: PROTOCOL: The effectiveness of sensory interventions targeted at improving occupational outcomes, quality of life, well‐being and behavioural and psychological symptoms for older adults living with dementia: A systematic review and meta‐analysis
Source: Campbell Syst Rev. 2023 Apr 10;19(2):e1322. doi: 10.1002/cl2.1322 (PMC10084746; doi:10.1002/cl2.1322)
Supplement: Supplementary file 1 — Supporting information. [file CL2-19-e1322-s001.docx]

# Appendices

## 1 Ovid Medline Search Strategy UPDATED

| **#** | **Searches** | **Results** | **Type** |
| --- | --- | --- | --- |
| 1 | exp dementia/ | 200087 | Advanced |
| 2 | (Dementia* or Alzheimer* or Creutzfeldt-Jakob or Huntington Disease or Kluver- | 271957 | Advanced |
|  | Bucy Syndrome or Lewy Body).ti,ab. |  |  |
| 3 | 1 or 2 | 308768 | Advanced |
| 4 | exp sensory art therapies/ or Relaxation Therapy/ or Muscle Stretching | 72796 | Advanced |
|  | Exercises/ or Gardening/ or Therapeutics/ or Aromatherapy/ |  |  |
| 5 | (Snoezelen* or multi-sensory* or acoustic or aromatherap* or ((sensory or | 618310 | Advanced |
|  | movement or art or colo?r or dance or music or play or creative or touch or |  |  |
|  | tactile or light) adj15 (integration or technique* or stimulat* or integrati* or |  |  |
|  | processing or intervention* or modul* or program* or class* or workshop* or |  |  |
|  | therap*)) or relaxation or stretch* or garden* or horticultur*).ti,ab. |  |  |
| 6 | 4 or 5 | 667757 | Advanced |
| 7 | "Quality of Life"/ or Adaptation, Psychological/ or exp "Activities of Daily Living"/ | 749062 | Advanced |
|  | or social behavior/ or exp aggression/ or cooperative behavior/ or help-seeking |  |  |
|  | behavior/ or social conformity/ or social inclusion/ or social isolation/ or social |  |  |
|  | skills/ or behavior/ or behavioral symptoms/ or exp impulsive behavior/ or exp |  |  |
|  | sleep wake disorders/ or exp "sleep initiation and maintenance disorders"/ or |  |  |
|  | obsessive behavior/ or paranoid behavior/ or problem behavior/ or wandering |  |  |
|  | behavior/ |  |  |
| 8 | (well-being or wellbeing or behavio?r or restless* or agitat* or irritat* or pacing or | 4337152 | Advanced |
|  | violen* or apathy or depress* or wander* or sleep* or abscond* or sundown* or |  |  |
|  | psycho* or adapti* or aggressi* or mood or social or symptom* isolation or |  |  |
|  | inclusion or participation or occupation or "Activities of Daily Living" or "quality of |  |  |
|  | life" or help-seeking or obsessive or paranoi* or problem or co-operative*).ti,ab. |  |  |
| 9 | 7 or 8 | 4555431 | Advanced |
| 10 | 3 and 6 and 9 | 2734 | Advanced |
| 11 | exp animals/ not humans.sh. | 5091864 | Advanced |
| 12 | 10 not 11 | 2632 | Advanced |
